# Complex biomembrane mimetics on the sub-nanometer scale

**DOI:** 10.1007/s12551-017-0275-5

**Published:** 2017-07-17

**Authors:** Frederick A. Heberle, Georg Pabst

**Affiliations:** 1 The Bredesen Center, University of Tennessee, Knoxville, TN 37996 USA; 2 Joint Institute for Biological Sciences and Biology and Soft Matter Division, Oak Ridge National Laboratory, Oak Ridge, TN 37831 USA; 3 Institute of Molecular Biosciences, Biophysics Division, NAWI Graz, University of Graz, 8010 Graz, Austria; 4 BioTechMed-Graz, 8010 Graz, Austria

**Keywords:** Asymmetric bilayers, Lipid domains, Lipid flip-flop, Lipid-protein interactions, Intermembrane interactions, Small-angle neutron and X-ray scattering

## Abstract

Biomimetic lipid vesicles are indispensable tools for gaining insight into the biophysics of cell physiology on the molecular level. The level of complexity of these model systems has steadily increased, and now spans from domain-forming lipid mixtures to asymmetric lipid bilayers. Here, we review recent progress in the development and application of elastic neutron and X-ray scattering techniques for studying these systems in situ and under physiologically relevant conditions on the nanometer to sub-nanometer length scales. In particular, we focus on: (1) structural details of coexisting liquid-ordered and liquid-disordered domains, including their thickness and lipid packing mismatch as a function of a size transition from nanoscopic to macroscopic domains; (2) membrane-mediated protein partitioning into lipid domains; (3) the role of the aqueous medium in tuning interactions between membranes and domains; and (4) leaflet-specific structure in asymmetric bilayers and passive lipid flip-flop.

## Introduction

Quantitative insight into biological processes is one of the major premises of biophysical research. To achieve this goal, it is often useful to reduce the level of biological complexity, for example by using simplified model systems, although the gained tractability needs to be carefully balanced with applicability to a given biological problem. Biological membranes are excellent examples for such endeavors. They are inherently complex mixtures of lipids, proteins, and carbohydrates that collectively make up a highly dynamic and flexible, yet well-structured, material. Membranes are not simply passive barriers for the compartmentalization of ‘inside’ and ‘outside’ processes; instead, they actively control transport or signaling events through or within membranes. Moreover, modern super-resolution microscopy has provided fascinating images showing that the interior of a eukaryotic cell is packed with membrane surfaces (Betzig et al. 2006; Huang et al. 2008; Shim et al. 2012), implying that most biochemical processes take place at membranes or in their immediate proximity. This puts biological membranes into center focus for drug research and novel therapies that aim to interfere with their physiological malfunction, or protect them from toxins or attack by pathogens (Pabst and Lohner 2014). Here, biophysics provides the tools to gain insight into some of the underlying fundamental processes. However, what is the most appropriate model of a cellular membrane?

Research on model lipid membranes goes back to the early 1960s, when Bangham and Horne used negative-stain electron microscopy to investigate liposomes made from lecithins dispersed in water (Bangham and Horne 1964). This was followed by the ‘golden age’ of lipid research, during which time several groups explored the physical chemistry of simple phospholipid-only membranes. However, molecular biological research then was focused almost exclusively on proteins, and did not take much notice of these developments. This situation was relatively unchanged until the late 1980s, when van Meer and Simons proposed a novel mechanism to explain how the apical and basolateral membranes of polarized epithelial cells acquire and maintain their unique lipid and protein compositions (Simons and Van Meer 1988). Their model posited that, within the trans Golgi membrane, nonrandom mixing of sphingomyelin and phosphatidylcholine lipids resulted in a micro-phase separation. Domains enriched in sphingolipids and cholesterol—later termed ‘lipid rafts’ (Simons and Ikonen 1997)—attracted a subset of Golgi-resident proteins which, after budding into transport vesicles, specifically targeted the basolateral membrane. Crucial evidence for the model followed, when it was shown that rafts could apparently be isolated from eukaryotic cells due to an unusual property: they were resistant to solubilization by non-ionic detergents such as Triton X-100 (Brown and London 1997). The raft hypothesis marked a turning point in biomembrane research, implying as it did that collective properties of membrane lipids are of considerable physiological relevance. Rafts are now thought to play a role in myriad cell phenomena including intracellular trafficking (Rajendran and Simons 2005), viral assembly and exit (Ono 2010), and cell signaling (Holowka et al. 2005). However, due to their apparently small size and dynamic nature, direct (i.e., visual) experimental evidence for the in vivo existence of membrane rafts is still controversial (Eggeling et al. 2009; Kraft 2013; Sevcsik et al. 2015).

In contrast, membrane domain formation is well established in lipid mixtures, especially those that mimic the composition of the exoplasmic leaflet of mammalian plasma membrane. As a general rule, these mixtures contain a minimum of three components: at least one lipid with a relatively high melting temperature (high-T_M_), such as sphingomyelin or long chain saturated phosphatidylcholines; at least one lipid with a relatively low melting temperature (low-T_M_), typically mono- or di-unsaturated or highly branched phosphatidylcholines; and cholesterol. Considerable effort has gone toward determining the various phase coexistence regions found in these mixtures (Veatch and Keller 2005; Feigenson 2009; Marsh 2009). Of particular interest is the composition- and temperature-dependent regime of coexisting liquid-ordered (Lo) and liquid-disordered (Ld) phases. After some initial controversy, compositional phase diagrams for these mixtures nowadays all agree that Ld domains are enriched in low-T_M_ lipids, and that Lo domains contain most of the high-T_M_ lipids and about 2–3 times more cholesterol than Ld (Marsh 2009). Thus, Lo domains are generally regarded as first-order mimics of membrane rafts. However, whether Lo domains in three-component model membranes faithfully reproduce the properties of membrane rafts, especially with regard to protein partitioning, is still an open question (Bacia et al. 2004; Kahya et al. 2005; Kaiser et al. 2011).

One of the most frequently heard criticisms of lipid-only studies is that the average protein mass of natural membranes is on the order of 60%, casting some doubt on the physiological relevance of these models. However, mass is not everything. A closer look at natural membranes reveals that most of a transmembrane protein’s mass is located either on the intracellular or extracellular side, so that only ~10–15% of matter bound by lipid headgroups is protein (Heberle and Feigenson 2011). This implies that the collective properties of the lipid matrix are indeed of significant importance to biological membranes. Clearly, a direct extrapolation of results from model membranes to live cells appears as a long, maybe too long stretch. Nevertheless, and in contrast to live cells, important fundamental properties of membranes can be explored under chemically and experimentally well-defined conditions using a huge repertoire of techniques, in order to explore their structural and dynamical nature at different length and time scales.

This review focuses on recent insight into complex lipid-only models obtained from elastic small-angle X-ray and neutron scattering (SAXS/SANS) from fully hydrated liposomal dispersions. Starting from the seminal work by the Luzzati laboratory on single-component lipid aggregates (Tardieu et al. 1973), both techniques have evolved significantly and are now able to tackle highly complex mimics of natural membranes. Here, we highlight recent progress made in our laboratories in the study of lipid domains of microscopic and nanoscopic size, as well as asymmetric lipid bilayers. SAXS and SANS allow the exploration of the terrain of membrane structure on the nano-to-subnanometer scale that is practically inaccessible to most other techniques, without resorting to bulky labels that might influence lipid phase behavior. The price to pay is heavy data modeling, as we discuss below. However, knowing that collective membrane properties on the mesoscopic scales couple directly to protein function and consequently cell physiology, makes a walk down that road certainly worthwhile.

## Cans and can’ts of small-angle scattering

SAXS or SANS experiments on lipid vesicles provide a global (statistical) average of membrane structure in both the lateral and transversal directions, originating from a contrast in scattering length density (SLD) due to a non-homogenous distribution of matter. This is complementary to other techniques, which provide insight on local (molecular) length scales and in particular either on the collective molecular dynamics [e.g., nuclear magnetic resonance (NMR), electron spin resonance (ESR), fluorescence spectroscopy, inelastic scattering, etc.], or single molecule behavior (e.g., lateral diffusion) for which lipophilic labels often need to be used. The accessible length scales of SAXS and SANS depend on the given experimental settings (sample-to-detector distance, wavelength, etc.), but in general range from ~0.1 to 100 nm. Elastic scattering experiments are therefore ideally suited for probing membrane structural details including bilayer thickness, average area per lipid molecule, and nanodomain size. Here, we provide only those aspects of both techniques which are important for grasping the essence of the reviewed results on complex membrane structures. For a recent detailed tutorial review, see Marquardt et al. (2015).

In order to ‘see’ structure on the nanoscale, our probe must have two essential properties: its wavelength must be of the size of the structures, and there must be significant contrast with respect to the bulk aqueous environment. Both neutrons and X-rays certainly have the proper wavelengths, but where does the contrast come from? X-rays and neutrons interact very differently with matter. X-rays are sensitive to the electron cloud of atoms, leading to high contrast between electron-rich and electron-poor regions of the material in question. For phospholipid bilayers, this translates into highly ‘visible’ polar headgroups due to the electron-dense phosphates. For neutrons, things are richer still, as neutrons interact with nuclei in a highly specific manner. Of particular importance for biological matter is the large difference in contrast between hydrogen (negative scattering length) and deuterium (positive scattering length), which enables the unique ability to make specific regions within a sample more visible as compared to others. In general, contrast variation in neutron experiments can be realized, either by manipulating the H_2_O/D_2_O content of the aqueous solution or by specific deuteration of the material (Jacrot 1976; Fitter et al. 2006). Indeed, the headgroup or acyl chain protons of phospholipids can be exchanged for deuterons to generate a large intramolecular contrast and thereby reveal the internal bilayer structure (Zaccai et al. 1975, 1979; Büldt et al. 1978; Léonard et al. 2001). It is important to realize that specific deuteration does not change the chemical nature of the material, which is a significant technical advantage as compared to the application of bulky lipid probes used in many spectroscopic techniques. In those cases, the signal must be carefully analyzed for potential probe-induced changes in the conformational equilibrium or partitioning (Heberle et al. 2014).

### Transbilayer structure

Despite the subtleties of their interaction with matter, the theory of scattering for X-rays and neutrons is fortunately analogous. Unfortunately, scattering means that—unlike imaging techniques—real space structural information cannot be obtained directly due to the loss of phase information. Instead, the real space information must be reconstructed. For lipid membranes, reconstruction can either be performed in a model-free way (Glatter 1977) or via the application of specific models (Heberle et al. 2012), depending on physical and chemical properties of the system. We focus here on the latter route, because it enables detailed structural insight into lipid systems with higher degrees of compositional flexibility, especially for membranes with inhomogeneity in both the lateral and transversal directions. We first focus on transbilayer structure.

**Fig. 1:**
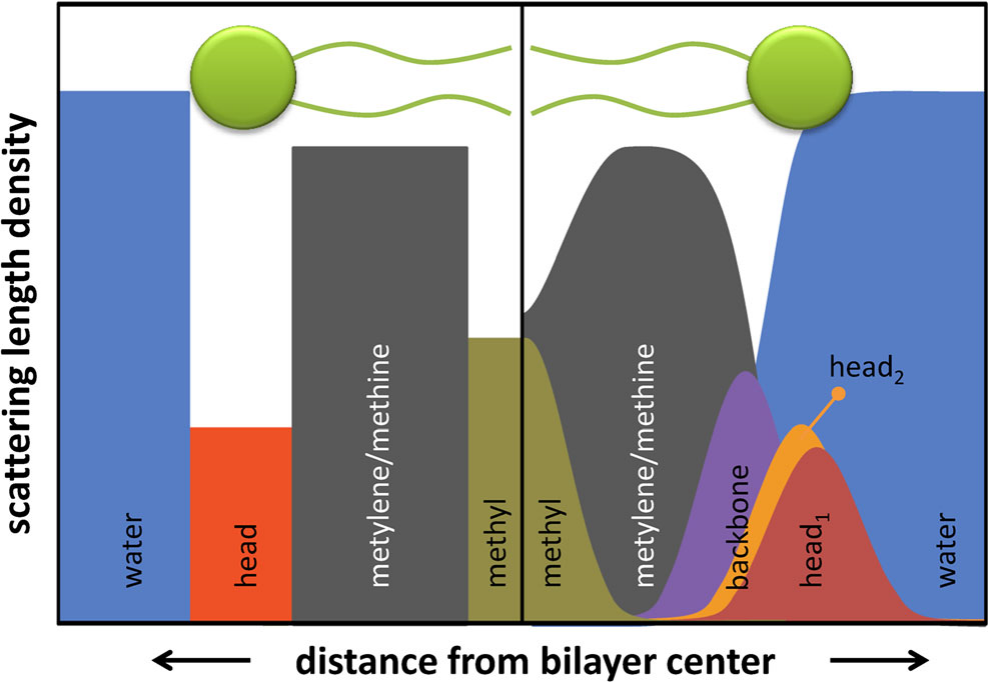
Scattering length density models with different detail for bilayer structure. The *left hand side *shows a slab model discriminating between the SLDs of lipid headgroup, hydrocarbon region and methyl terminus of the hydrocarbon chains. The *right hand side *resembles the SDP model, which considers distribution functions of Gaussian type for quasi-molecular fragments of lipid molecules. Typically additional groups, such as, e.g., the glycerol backbone or a splitting of the headgroup (into phosphate and primary alcohol groups) are considered

Over the years, diverse models for the transbilayer structure have been reported (Nagle and Tristram-Nagle 2000; Pabst et al. 2010; Heberle et al. 2012; Fogarty et al. 2015). The simplest of these is to divide the bilayer structure into slabs of different SLD, where the number of slabs depends on the resolution of both the experiment and the bilayer itself (Fig. 1). For example, for a protiated lipid bilayer in D_2_O, there is insignificant neutron contrast between the hydrocarbon terminal methyl groups and the methylene groups. Hence, it is usually sufficient to consider only two slabs corresponding to the headgroup and hydrocarbon regions. However, sharp boundaries between slabs do not appear to be realistic in view of dynamic motions of the individual lipid molecules. The scattering density profile (SDP) model accounts for the transverse positional fluctuations by parsing the lipid’s constituent atoms into quasi-molecular fragments, whose volume probabilities are described mathematically by distribution functions (Klauda et al. 2006; Kučerka et al. 2008). Parsing is achieved with the aid of molecular dynamics (MD) simulations, which greatly facilitate the comparison of distribution profiles for different proposed atomic groupings. SDP models have been reported for phosphatidylcholine (Kučerka et al. 2011), phosphatidylglycerol (Pan et al. 2014a), phosphatidylethanolamine (Kučerka et al. 2015), phosphatidylserine (Pan et al. 2014b), ether-linked phosphatidylcholine (Pan et al. 2012), and cardiolipin (Pan et al. 2015). The primary advantage of the SDP model compared to simpler slab models is in the resolution of the internal bilayer structure. However, this needs to be carefully counterbalanced against the danger of overparameterization.

Both the slab and SDP models have been successfully used to derive the lateral area per lipid *A*_*L*_, an important parameter for understanding the lipid packing density in membranes, by requiring ideal space filling of the lipid molecules. This assumption couples the molecular volume *V*_*group*_ of each slab or quasimolecular distribution function and its position with respect to the bilayer center *z*_*group*_, from which *A*_*L*_ = *V*_*group*_/*z*_*group*_ emerges as a fitting parameter. Bilayer thickness parameters, including the hydrocarbon chain thickness, the headgroup-to-headgroup thickness, and the Luzzati thickness are consequently given by different combinations of *z*_*group*_. Lipid volumes are supplied by independent densitometry measurements (Nagle and Tristram-Nagle 2000; Koenig and Gawrisch 2005; Greenwood et al. 2006; Uhríková et al. 2007; Klacsová et al. 2010; Murugova and Balgavý 2014; Miyoshi et al. 2014).

Highest structural confidence can be obtained by taking advantage of the different contrasts probed by X-rays and neutrons, including the above-mentioned isotope labeling options and H_2_O/D_2_O exchange. A joint analysis of SANS and SAXS data couples all these different contrasts into one structural model, which is a reliable way to reduce the pitfalls of potential overparameterization of the SDP analysis (see, e.g., (Kučerka et al. 2011; Heftberger et al. 2014). In fact, this also allows differentiation between the individual structural leaflet properties of asymmetric membranes (Heberle et al. 2016; Eicher et al. 2017), as described below. A different degree of complexity is found in lipid vesicles displaying coexisting Lo/Ld domains. In this case, each domain is composed of at least two phospholipid species and cholesterol. Certainly, one could attempt to model each lipid species individually; however, this would lead to an inordinate number of adjustable parameters. An appropriate way to handle such issues is the use of molecular averages of the individual lipid properties, thus defining a virtual hybrid molecule, which is then used as the unit cell in the structural analysis (Heftberger et al. 2015; Belička et al. 2017). The individual slabs/quasimolecular distributions thus represent a weighted composite of the individual lipid species. In this way, it has even been possible to analyze coexisting Lo/Ld domains in situ, gaining insight into structural subtleties including their cholesterol content (Belička et al. 2017). We will illustrate this in an example given below.

### Lateral structure

**Fig. 2:**
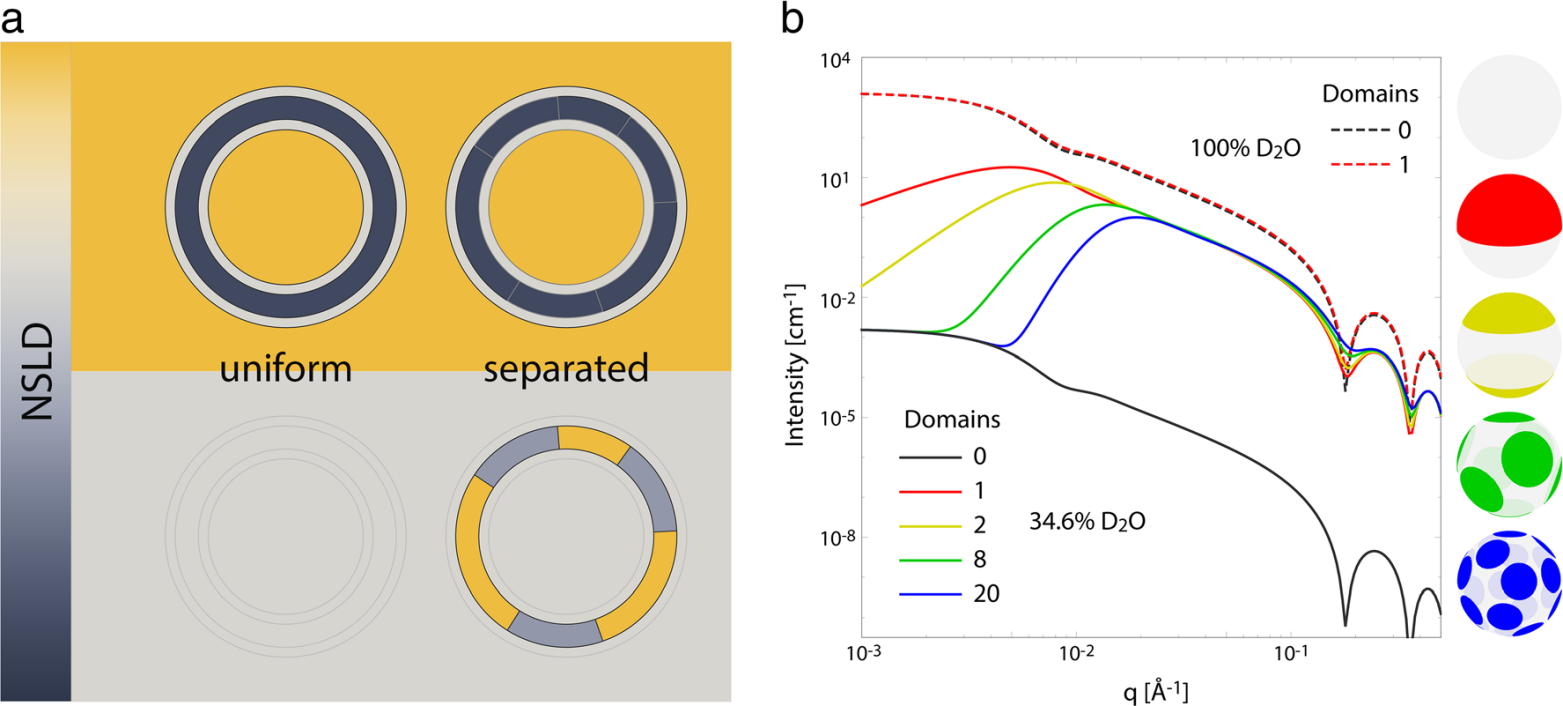
Lateral organization of membranes investigated with neutron scattering. **a** Schematic illustration of selective lipid deuteration and solvent contrast matching to facilitate the detection of membrane domains. **b** Calculated scattering curves for vesicles with the same average bilayer NSLD, but under different conditions of solvent NSLD and/or lipid lateral organization. Scattering curves were calculated with an analytical form factor for phase-separated vesicles described in Heberle et al. (2015), using 100 expansion orders and model parameters stated in the main text. Figure adapted from Marquardt et al. (2015)

In phase-separated membranes, differences in the composition of coexisting domains results in a lateral SLD contrast, in addition to the usual transverse contrast between bilayer and solvent discussed in the previous section. Typically, the in-plane contrast is vastly smaller than the transverse contrast, and lateral structure contributes negligibly to the scattering intensity, as shown schematically in the upper panel of Fig. 2a. However, experimental conditions can often be optimized to suppress transverse contrast and enhance lateral contrast (Pencer et al. 2006). This is especially true for neutron scattering, where the availability of D_2_O- and deuterium-labeled lipids provides a straightforward means of manipulating both the solvent and membrane neutron SLD (NSLD) without perturbing the bilayer structure. By judicious mixing of protiated and deuterated lipid species, it is often possible to simultaneously match the average NSLD of the lipid headgroup and acyl chain regions to the surrounding water, as shown in the lower panel of Fig. 2a. If under these conditions the lipids are randomly mixed, no transverse or lateral contrast exists in the sample volume, resulting in a null scattering condition. However, if the protiated and deuterated lipid species instead segregate from each other, the resulting in-plane contrast gives rise to a coherent scattering signal. Indeed, observation of an abrupt increase in scattering with decreasing temperature has convincingly demonstrated phase coexistence in biomimetic mixtures containing cholesterol (Pencer et al. 2005; Masui et al. 2008; Vogtt et al. 2010; Heberle et al. 2013a, b; Petruzielo et al. 2013), without the need for extrinsic fluorophores or spin-labeled lipids.

Scattering curves obtained from samples that have been optimized for lateral contrast contain valuable information about domain size, shape, and configuration, encoded within the scattering vector (*q*)-dependence of the scattered intensity (*I*). However, models for quantitatively interpreting *I*(*q*) have lagged behind simpler model-free analyses such as the forward scattering method (Knoll et al. 1981) and the Porod invariant (Pencer et al. 2006), which primarily yield qualitative information about the presence or absence of phase separation in a sample. Real-space Monte Carlo (MC) methods based on the Debye formula (see, e.g., Henderson 1996) have successfully been used to model scattering from complex domain morphologies that are otherwise intractable analytically, but a drawback of this approach is that it is more difficult to use in a nonlinear-least squares fitting routine. Analytical approximations for the domain form factor (e.g., as a cylindrical disk) are more useful in this regard, and have been employed to model scattering data from heterogeneous vesicles, although without accounting for the spherical shape of the vesicle itself, or any contributions from a multi-domain structure factor (Vogtt et al. 2010). A more recent analytical approach combined the form factor for a perforated, infinitely thin, spherical shell with that of a flat lipid bilayer, thereby conceptually replacing the solvent-filled holes in the shell with disk-like lipid domains (Dao et al. 2017). This ‘holey shell’ form factor was used to model scattering data from hybrid polymer/lipid vesicles to recover domain sizes.

Anghel and coworkers were the first to provide a general and exact analytical solution for a laterally heterogeneous lipid vesicle (or more generally, core-shell particle) (Pencer et al. 2005; Anghel et al. 2007). The solution was expressed as a spherical harmonic expansion of the scattering amplitude for a three-shell vesicle (i.e., one shell each for the headgroup and hydrocarbon slabs, as in Fig. 2a) containing a single round domain, and was successfully used to model experimental data from binary mixtures of dipalmitoyl phosphatidylcholine (DPPC) and dilauroyl phosphatidylcholine (DLPC) (Anghel et al. 2007). The single-domain form factor was, however, found to be inadequate to describe scattering from three-component mixtures of DPPC, dioleoyl phosphatidylcholine (DOPC), and cholesterol (Chol) that are known to separate into coexisting Ld and Lo domains (Pencer et al. 2005). Instead, an MC analysis was consistent with multiple smaller domains in these vesicles (Pencer et al. 2005; Masui et al. 2008).

More recently, these authors extended the spherical harmonic expansion to the case of multiple round domains (Heberle et al. 2015). In this case, the scattering intensity consists of three terms, i.e. *I*(*q*) = *I*_hom_(*q*) + *I*_intra_(*q*) + *I*_inter_(*q*). The homogeneous term *I*_hom_ accounts for the transverse bilayer structure of each phase and is minimized when the NSLD of the headgroup and hydrocarbon layers is matched to that of the solvent. The last two terms comprise the heterogeneous contribution to the intensity and represent the single domain form factor *I*_intra_ and multi-domain structure factor *I*_inter_ originating from domain–domain in-plane cross-correlations; these terms are expressed as an infinite sum over spherical harmonics. As few as 20 expansion orders were required to capture details of the scattering at intermediate length scales of 5–100 nm, as judged by a comparison to MC simulated data.

Figure 2b shows an illustrative demonstration of the analytical model for different conditions of solvent contrast and lipid lateral organization. To approximate experimental conditions (e.g., Heberle et al. 2013a, b), the calculations assume 60-nm-diameter vesicles with 30% relative polydispersity, at 1 wt% lipid. It is further assumed that the domain and surrounding phases each occupy half the total vesicle surface area, and have different NSLDs (0.32 and 0.04 fm Å^−3^, respectively) caused by nonuniform partitioning of a selectively deuterated lipid. The average bilayer NSLD is in every case 0.18 fm Å^−3^, such that the observed differences in I(q) result from differences in either the solvent NSLD or the domain organization. For vesicles in D_2_O (NSLD 0.636 fm Å^−3^), *I*_hom_ dominates the scattering due to the large contrast between the solvent and bilayer. In this case, the scattering curve of a phase-separated vesicle (red dashed line) cannot be visually distinguished from that of a uniformly mixed vesicle (black dashed line). However, at solvent/bilayer contrast matching conditions (34.6% D_2_O, NSLD 0.181 fm Å^−3^), *I*_hom_ is dramatically attenuated (black solid curve), and the effects of the domain structure factor are more pronounced (colored solid curves corresponding to schematic vesicle images). In principle, the same analytical form factor can also be used to model compositional fluctuations at smaller length scales typically associated with highly non-ideal mixing as opposed to true phase separation (Heberle and Feigenson 2011; Ackerman and Feigenson 2015), although hundreds of expansion orders (and considerable computational effort) may be required in such a case.

### Interactions

An interesting variant of SAXS experiments on liposomal dispersions are so-called osmotic stress (OS) experiments, pioneered by Parsegian and Rand (LeNeveu et al. 1976). These experiments allow the exploration of forces between membranes (and other macromolecules) and their modulation by properties of the aqueous phase. Such information is highly relevant to biological processes in view of the tightly packed space in cells mentioned above.

**Fig. 3:**
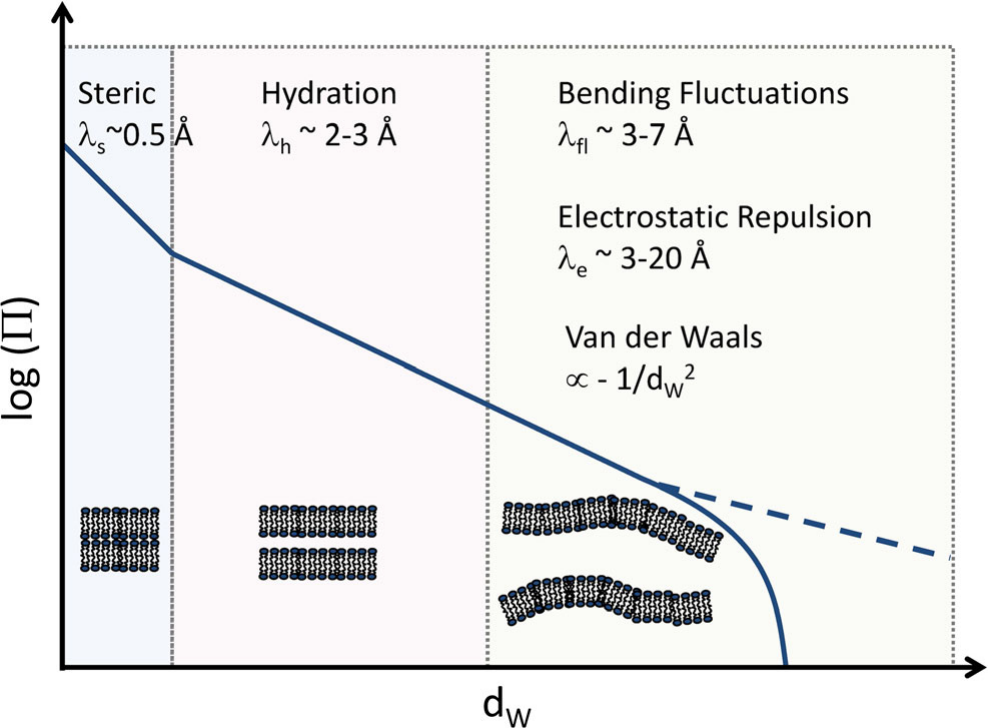
Generic dependence of bilayer separation d_W_ under osmotic pressure. The pressure isotherm exhibits essentially three regimes, where different forces dominate or compete with each other. Typical length scales for the exponential decays and schematics for lipid bilayers are given. The *dashed line *indicates highly repulsive bilayers that do not attain a finite separation in the absence of osmotic pressure

To date, the most widely used way of applying osmotic pressure to liposomal dispersions is through large neutral polymers, such as polyethylene glycol, that are excluded from the interstitial water layers and lack direct interactions with the bilayers. Raising osmotic pressure by increasing the bulk polymer concentration effectively reduces the distance between adjacent membranes, which is directly probed by SAXS. Lipid membranes, subjected to osmotic stress, reveal exponential variation of opposing forces with bilayer separation, which can be (depending on the dominant interaction at a given pressure) roughly dissected into three different regimes (Fig. 3): (1) steric interactions at dry “contact” due to headgroup collisions, (2) hydration interactions at intermediate separations thought to be due to the work of polar group dehydration, sometimes additionally enhanced by lamellar collisions from thermal agitation (Parsegian et al. 1994); and (3) the highly swollen regime which balances repulsive interactions due either to bending fluctuations (Helfrich 1978), and/or electrostatic interactions, and to attractive Van der Waals forces (Parsegian 2004). Analysis of the pressure isotherms therefore allows for a deconvolution of the contributions of the individual forces to interbilayer interactions. We note, however, that this analysis easily becomes non-trivial (especially for flexible bilayers) because bending fluctuations, due to their entropic origin, couple to all other forces and lead to their renormalization. Further below, we illustrate two recent applications of this technique. For general reviews on OS, see, e.g., Parsegian and Rand 1995; McIntosh 2000; Petrache et al. 2007; Harries and Raviv 2014).

Besides these direct measurements of interactions between adjacent membranes, scattering techniques also provide indirect insight into interactions with transmembrane proteins, especially those mediated by membrane elastic and structural properties. Such membrane-mediated interactions may be due either to differences in the hydrophobic stretches of proteins and lipid bilayers (i.e., hydrophobic matching) (Dan et al. 1993; Marsh 2008a; Lundbæk et al. 2010), or to coupling with composition-dependent lateral pressure profiles (Cantor 1997, 1999; Dan and Safran 1998). Here, we focus on the latter mechanism, because it can be readily explored using scattering experiments.

**Fig. 4:**
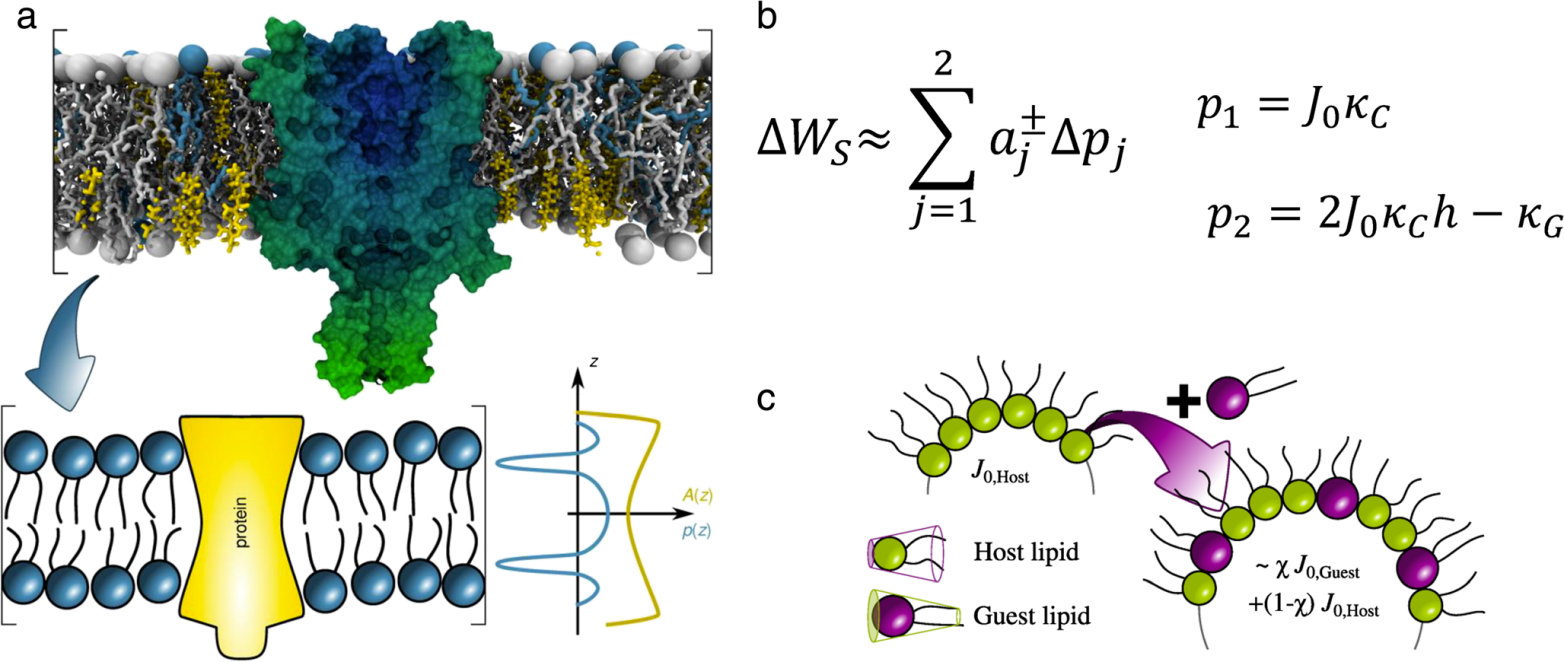
Lateral pressure-mediated effects on transmembrane proteins. **a** Schematic illustration of the transformation of a membrane protein in a complex lipid environment, into a protein with simple geometric shape in a homogeneous symmetric lateral pressure field. First-order lateral pressure-mediated contributions to transmembrane protein partitioning into a given lipid environment (e.g., Lo/Ld domains) can be expressed by coefficients *a*_j_^±^ that describe protein shape and the first and second moments of lateral pressures *p*_*j*_ (*J*_*0*_…intrinsic curvature, *κ*_*C*_ *…*monolayer bending rigidity, *h*… position of the neutral plane, *κ*_*G*_ …Gaussian modulus of curvature) ( **b**) (Frewein et al. 2016). **c** The principle of estimating *J*_*0*_, in which template lipids forming inverted hexagonal phases (such as dioleoyl phosphatidylethanolamine) are doped with guest lipids of unknown intrinsic curvature. The intrinsic curvature of the guest lipid is determined from a series of scattering experiments assuming linear additivity of the individual lipid curvatures. Figure adapted from Kollmitzer (2015)

Depth-dependent lateral pressures in membranes originate from a balance of attractive pressures due to minimizing the exposure of hydrophobic entities at the polar/apolar interface and repulsive pressures on either side of this boundary. These repulsive contributions are dominated by electrostatic interactions for lipid headgroups and by entropically-driven fluctuations for hydrocarbons (Ben-Shaul 1995). A protein inserted into such a pressure field will minimize its free energy with respect to its function and/or its location within a given lipid environment. This may result in membrane-mediated contributions to protein activity (e.g., channel formation), clustering or preferential partitioning into membrane domains. Lateral pressure profiles are difficult to determine experimentally (Templer et al. 1998), but can be derived from molecular dynamics (MD) simulations (Ollila and Vattulainen 2010). Yet, it is possible to gain some experimental insight by approximating transmembrane proteins with simple geometric shapes (Fig. 4). The energetic contributions resulting, for example, from physicochemical differences of Lo/Ld domains can be related to membrane parameters, such as the intrinsic curvature, monolayer bending rigidity, Gaussian modulus of curvature, and location of the neutral plane, which can be all determined or estimated from scattering experiments (Pabst 2013; Kollmitzer et al. 2013; Frewein et al. 2016). We present results from such a study in “Membrane-mediated coupling to transmembrane proteins”.

## Lateral complexity in membranes (domains/lipid rafts)

The first report of coexisting Lo/Ld domains in model membranes by fluorescence microscopy (Dietrich et al. 2001) was a kickoff for intense research efforts by diverse groups aiming to describe the biophysical properties of these first-order mimics of membrane rafts. Soon, significant controversies came up regarding the composition of Lo/Ld domains in ternary lipid mixtures (Marsh 2009). About a decade later, however, the major issues were resolved, and today there is consensus that Lo domains contain most of the high-T_M_ lipids and ~2–3 times more cholesterol than Ld domains, which are enriched in the low- T_M_ lipid component (Heberle et al. 2014). Various properties of coexisting domains have been studied, including critical fluctuations at the transition point to homogeneously mixed membranes (Veatch et al. 2007). Here, we focus specifically on domain size as a function of lipid composition and the corresponding transmembrane structures as determined by SANS and SAXS.

### Domain size

One of the most controversial aspects of the lipid raft hypothesis concerns the size of rafts in the plasma membrane. Much of the early evidence for rafts in cells came not from direct visualization of domains but rather from biochemical assays—most notably the presence in cell membrane extracts of a detergent resistant fraction with Lo-like composition (Brown and London 1997)—that were later found to be prone to significant artifacts (Heerklotz 2002; Hancock 2006; Brown 2006). This led to intense effort in the 2000s to develop a new and robust methodology for probing lateral heterogeneity at the nanoscale (Elson et al. 2010). Model membrane studies led the way in this effort, with reports of nanoscopic phases in three-component mixtures detected by Förster resonance energy transfer (FRET) (Feigenson and Buboltz 2001; De Almeida et al. 2003), ESR (Chiang et al. 2004; Smith and Freed 2009; Ionova et al. 2012), fluorescence quenching (Pathak and London 2011), and neutron scattering (Nicolini et al. 2004; Pencer et al. 2005). In more recent years, advanced imaging techniques including super-resolution microscopy (Eggeling et al. 2009) and high-resolution imaging mass spectrometry (Frisz et al. 2013) have provided direct evidence of lateral heterogeneity in cell membranes. Current research efforts in model membranes are focused on understanding the nature of small domains in cells, including mechanisms for raft formation and control of raft size at the level of lipid–lipid and lipid–protein interactions. As we discuss below, a wide range of domain sizes and morphologies are accessible in mixtures containing as few as four lipid components.

**Fig. 5:**
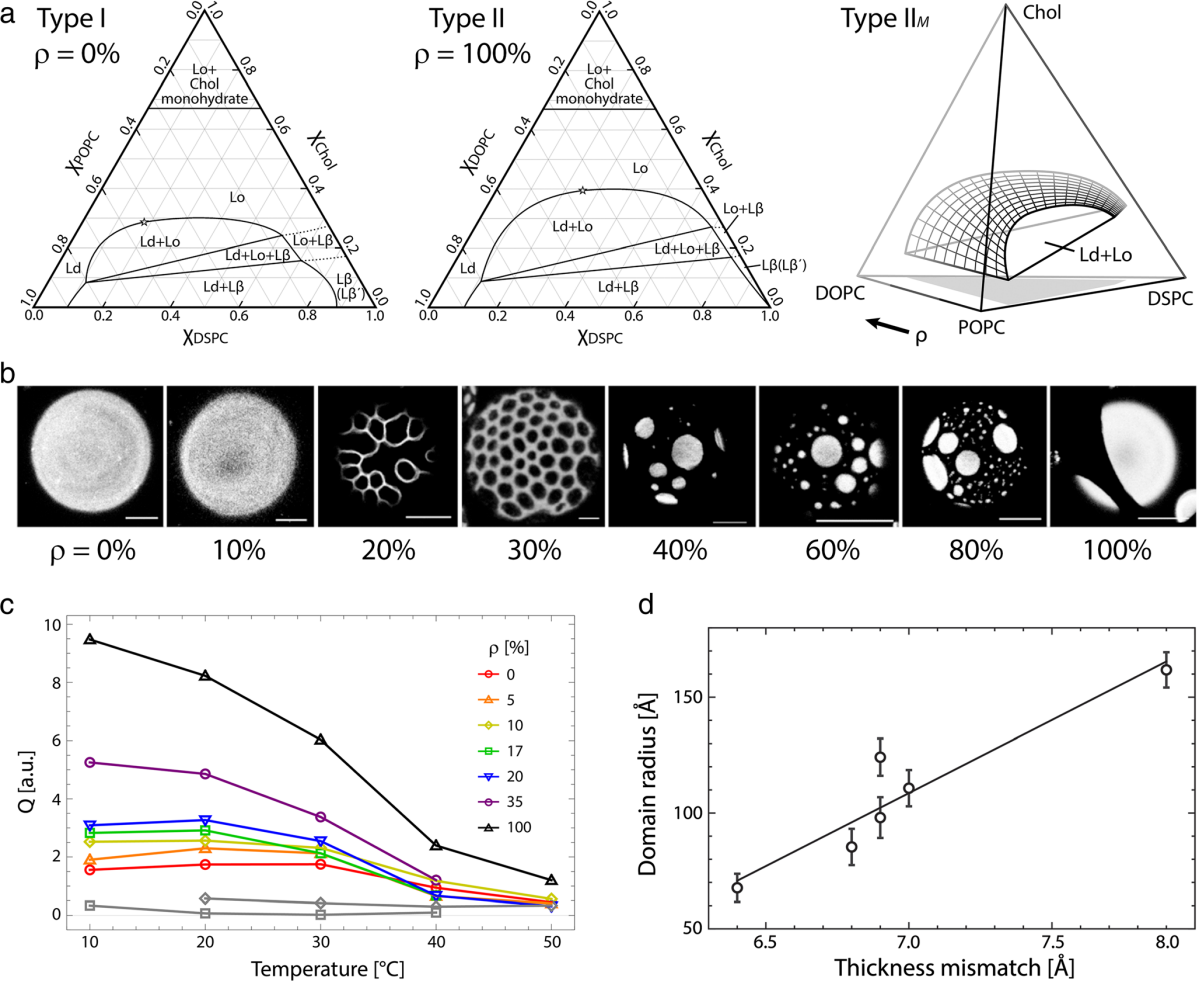
Domain size transitions in four-component model systems. **a** Three-component phase diagrams for the Type I mixture DSPC/POPC/Chol (*left*) and the Type II mixture DSPC/DOPC/Chol (*center*) provide two faces of a tetrahedral phase diagram for the four-component mixture DSPC/DOPC/POPC/Chol (*right*). The parameter ρ describes the fraction of DOPC to total low-melting lipid. **b** Fluorescence images of giant unilamellar vesicles at 23 °C with composition DSPC/(DOPC + POPC)/Chol = 39/39/22 mol% reveal a domain size transition from uniform appearance (ρ < 20%) to modulated phase patterns (20 < ρ < 40%) to large round domains (ρ > 40%). *Scale bars *10 μm. **c** Total integrated small-angle neutron scattering intensity versus temperature for 60-nm-diameter unilamellar vesicles with composition DSPC/(DOPC + POPC)/Chol = 39/39/22 mol%, for different values of ρ as indicated in the figure legend. Single phase control samples are shown in *gray*. **d** Domain radius versus thickness mismatch obtained from modeling the SANS data. Figure adapted from Heberle et al. (2014), Marquardt et al. (2015) and Usery et al. (2017)

Feigenson and coworkers pioneered the use of four-component mixtures for investigating domain size transitions. This work began with the observation that phase diagrams for three-component mixtures consisting of a low-T_M_ lipid, a high-melting high-T_M_ lipid, and cholesterol exhibited two categories of behavior with respect to the Ld + Lo coexistence region (Fig. 5a). In Type II mixtures, coexisting micron-sized domains are observed in fluorescence microscopy experiments, and this type of behavior is found for low-T_M_ lipids including DOPC (Zhao et al. 2007), diphytanoyl phosphatidylcholine (DPhPC) (Veatch et al. 2006), and 18:0,22:6-PC (Konyakhina and Feigenson 2016). Such domains are also frequently referred to as macroscopic domains. In Type I mixtures, Ld + Lo domains are not seen with optical microscopy, but can nevertheless be detected with techniques like FRET, ESR, and SANS that have nanoscale sensitivity (nanoscopic domains); this type of behavior is found for the low-T_M_ lipids palmitoyl oleoyl phosphatidylcholine (POPC) (De Almeida et al. 2003; Heberle et al. 2010), stearoyl oleoyl phosphatidylcholine (SOPC) (Heberle et al. 2010), and DLPC (Feigenson and Buboltz 2001; Heberle et al. 2013b). Despite the differences in domain size, the phase diagrams for Type I and Type II mixtures are similar and contain the same phase-coexistence regions (Feigenson 2009).

Konyakhina et al. determined the quaternary (tetrahedral) phase diagram for the mixture distearoyl phosphatidylcholine (DSPC)/DOPC/POPC/Chol, finding that the Ld + Lo region is continuous within the four-component space as one low-T_M_ lipid is replaced by the other, as shown in the rightmost panel of Fig. 4a (Konyakhina et al. 2013). In other words, starting from an Ld + Lo composition in DSPC/DOPC/Chol and replacing the Type II lipid DOPC with the Type I lipid POPC, a domain size transition from microns to nanometers occurs without crossing a phase boundary, as shown in the giant unilamellar vesicle (GUV) images in Fig. 5b. Remarkably, the large round domains characteristic of Type II mixtures do not appear suddenly as composition is varied; rather, the first optically resolvable domains appear as thin stripes with distinctive spatial modulation (Konyakhina et al. 2011). Modulated phase regions have been observed in at least 13 different mixtures (Usery et al. 2017), and appear to be a general phenomenon in four-component mixtures of the type high-T_M_/Type I low-T_M_/Type II low-T_M_/cholesterol, referred to as Type II_*M*_ mixtures (Heberle et al. 2014). Modulated domain patterns are characteristic of competing molecular interactions favoring very different length scales (Seul and Andelman 1995). In lipid bilayers, line tension at the domain edge favors large domains (Esposito et al. 2007), while mismatches in the material properties of the coexisting phases (e.g., bending energy or dipole density) favors small domains (Amazon et al. 2013; Amazon and Feigenson 2014; Usery et al. 2017). Modulated domains are the equilibrium outcome when the competing interactions are roughly balanced.

The nanoscopic regime of the domain size transition has been examined in greater detail by Heberle et al. using SANS together with global contrast matching as described in a previous section, as shown in Fig. 5c, d (Heberle et al. 2013a). Scattering curves for mixtures containing DSPC/(DOPC + POPC)/Chol (39/39/22 mol%) at 20 °C were fit using model curves obtained from MC simulation of contrast-weighted pair–distance distributions assuming monodisperse, round domains (Pan et al. 2013). As POPC was gradually replaced by DOPC, the domain radius increased from 6.8 to >16.5 nm. At the same time, the thickness mismatch between the coexisting Ld and Lo phases increased from 0.64 to 0.97 nm, consistent with the notion of increasing line tension driving domain coalescence (Fig. 5d). We note that the upper limit for domain sizes is dictated by vesicle size (i.e., the largest domain size detectable will follow the sequence LUV < MLV < GUV).

### Transbilayer structural differences between Lo and Ld domains

**Fig. 6:**
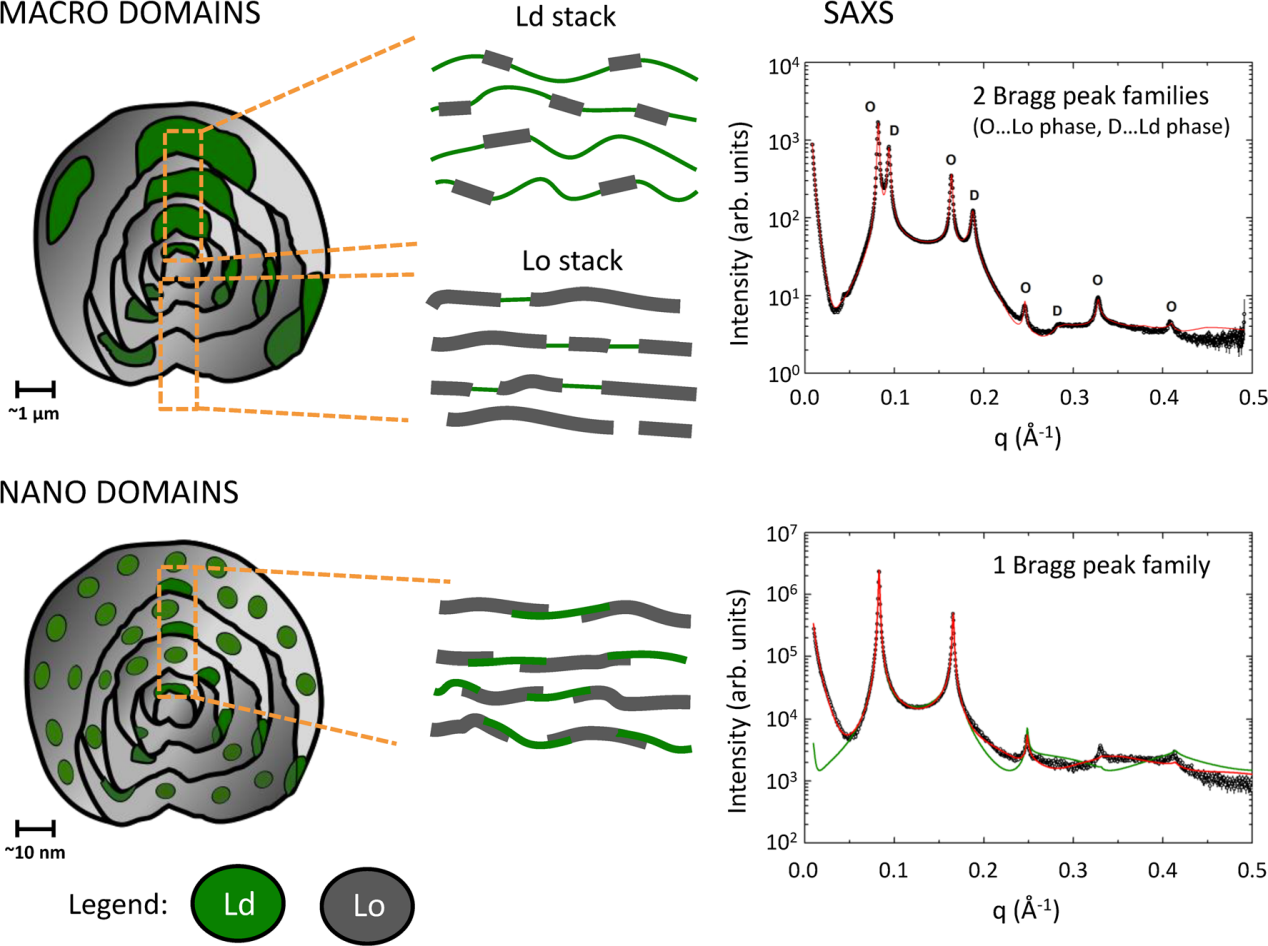
Macroscopic and nanoscopic domains in MLVs, in DSPC/DOPC/Chol = 30/46/24 mol% and DSPC/POPC/Chol = 39/39/22 mol%, respectively, at 20 °C. Macroscopic domains show long-range alignment of like domains leading to SAXS pattern with two lamellar lattices. The model fit (*red solid line*) also considers contributions from positionally uncorrelated Lo domains in Ld stacks and vice versa. SAXS data of nanoscopic domains show only a single lamellar lattice signifying the absence of domain alignment. The underlying model (*red solid line*) considers partial overlap of Lo and Ld domains. A homogenous (ideally mixed) model of membrane structure was unable to fit experimental data. Data taken from Belička et al. (2017)

Atomic force microscopy (AFM) first demonstrated that Lo domains are significantly thicker than coexisting Ld domains (see, e.g., Connell and Smith 2006, for review). This finding agreed with early X-ray studies that isolated Lo from Ld domains by application of the detergent Triton X-100 (Gandhavadi et al. 2002), as well as a recent SANS study that focused on tieline endpoints (Heberle et al. 2013a). The analysis of scattering data from phase-separated membranes was slower to develop, due to the intricacy of deconvoluting the signal into separate contributions from Lo and Ld. Heftberger and coworkers were the first to describe an in situ analysis of SAXS data from coexisting Lo/Ld domains at the accuracy of the SDP model (Heftberger et al. 2015). The analysis relies strongly on the fact that coexisting macroscopic domains are typically aligned three-dimensionally in multilamellar systems (Tayebi et al. 2012). In scattering experiments on multilamellar vesicles (MLVs), this leads to the observation of two well-separated families of lamellar Bragg peaks, which can be assigned to either the Lo or Ld phase, and which can be modeled considering their line-shapes (Zhang et al. 1994; Pabst et al. 2000) (Fig. 6).

**Fig. 7:**
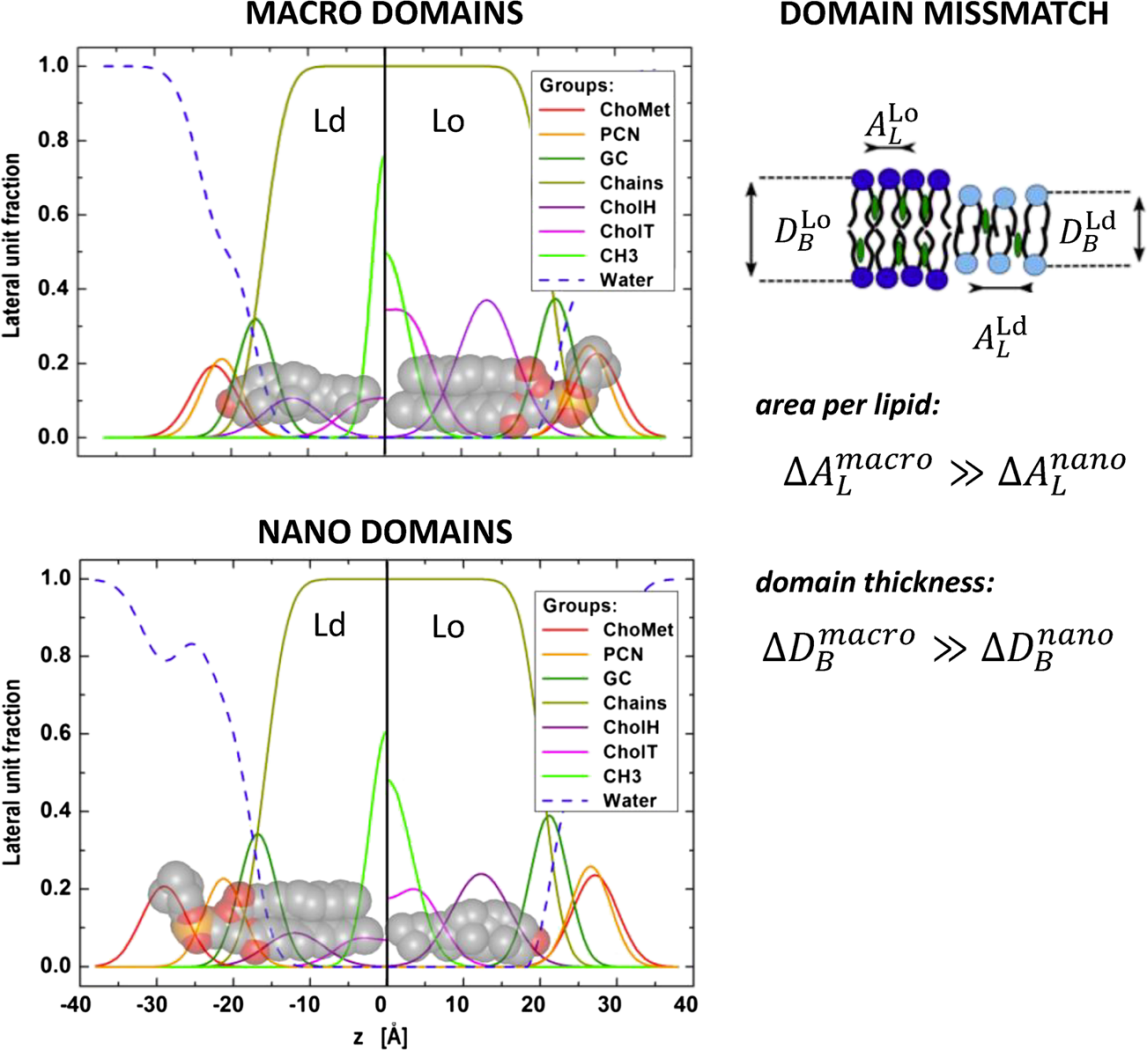
Transbilayer structural details of coexisting Lo/Ld domains in the macroscopic (*top*) and nanoscopic regime (*bottom*). The mixtures were composed of DSPC/DOPC/Chol (macroscopic domains) and DSPC/POPC/Chol (nanoscopic domains). Figure adaped from Belička et al. (2017)

Most recently, we can further refine the modeling by taking into account contributions from unlike domains within a given stack of like domains (Belička et al. 2017). A detailed analysis of internal domain structure showed a thickness mismatch of ~1.2 nm between the Lo and Ld domains in a ternary mixture of DSPC/DOPC/Chol, and a concomitant area per lipid mismatch of ~0.12 nm^2^ (Fig. 7). Hence, Lo domains are not only significantly thicker than Ld domains but also much more tightly packed. This is attributed to the lateral condensing effect of cholesterol and its preferred interaction with saturated lipids (McConnell and Radhakrishnan 2003; Pan et al. 2009b). Both cholesterol and saturated lipids are enriched in Lo domains. An additional feature of the refined analysis of coexisting domains is the ability to determine the relative molar ratio of cholesterol in each domain directly from the fit. For DSPC/DOPC/Chol, the determined values r_Lo_ = 0.493 and r_Ld_ = 0.208 were in excellent agreement with those reported independently from FRET experiments (Heberle et al. 2010).

While long-range alignment of like domains in MLVs was dominant for Type II mixtures, nanosopic domains apparently lose the ability of ‘self-alignment’. This is most clearly expressed by the observation of only a single lamellar lattice for DSPC/POPC/Chol (Fig. 6). Yet, the recorded scattering does not correspond to that of a homogenous membrane, as demonstrated by the inability of such a model to account for experimental data. The successful model described the MLVs with a single lamellar lattice, with each layer being composed of freely floating Lo, Ld and fractions of partially overlapping Lo/Ld (i.e., leaflet anticorrelated) domains, which do not positionally correlate to domains in adjacent layers (Belička et al. 2017). Interestingly, the derived details of transbilayer structure for DSPC/POPC/Chol (Fig. 7) showed a decrease of structural mismatch not only for domain thicknesses in agreement with SANS experiments (Heberle et al. 2013a) but also for lipid packing. In particular, the differences between Lo/Ld thicknesses decreased to ~1 nm, and the area per lipid mismatch to ~0.08 nm^2^. In other words, Lo and Ld domains become more alike, which is mainly achieved by corresponding modifications of the Ld structure. This could be either due to the different lipid composition of Ld domains or to the effects from domain size, an issue which still needs to be explored. Differences in quantitative values for thicknesses between SAXS (Belička et al. 2017) and SANS (Heberle et al. 2013a) experiments relate to the different details of applied SLD models. We note that the observation of a single lamellar lattice for coexisting nanoscopic domains might also be influenced by the coherence length of the X-ray beam, which, if significantly larger than the positional correlations between like-domains, can average out the signal of their corresponding individual lattices (see, e.g., Armstrong et al. 2012; Marquardt et al. 2015; Belička et al. 2017)]. The alignment of like-domains across the aqueous phase itself poses interesting questions regarding the origin of this coupling, which will be discussed in “Coupling through the aqueous phase”.

## Transbilayer complexity in membranes (asymmetry)

With few exceptions, biological membranes are asymmetric. For example, within the mammalian plasma membrane nearly all of the high-melting sphingomyelin (SM) lipids are found in the outer leaflet, while phosphatidylethanolamine (PE) and the negatively charged lipids phosphatidylserine (PS) and phosphatidylinositol (PI) are almost completely segregated in the inner leaflet (Devaux and Morris 2004). This asymmetry is not an accident of evolution; cells expend valuable energy to create and maintain it (Daleke 2003). Indeed, one of the first markers of impending cell death is the appearance of PS lipids in the plasma membrane outer leaflet, where they do not normally reside (Schlegel and Williamson 2001). Although the existence of asymmetry was first demonstrated over 40 years ago (Bretscher 1972), surprisingly little is known about its structural and functional consequences, nor how the properties of the two leaflets are coupled. A first and crucial step toward understanding how the cell uses asymmetry is to develop a picture of the physicochemical properties of asymmetric bilayers, but much of what is known about lipid membrane structure and dynamics has come from the study of *symmetric* model bilayers. It has historically been difficult to prepare and purify asymmetric liposomes, let alone to accurately measure the composition of the individual leaflets, a prerequisite for developing testable predictions about coupling mechanisms. Technical shortcomings have therefore largely precluded studies of asymmetric membranes as a function of, e.g., leaflet composition. However, recent advances in the field, described below, have now paved the way for such systematic studies.

### Model systems for studying asymmetry

It is difficult to systematically study asymmetry in natural cell membranes because of their chemical complexity. A variety of model systems have therefore been conceived to examine asymmetry in simpler lipid bilayer mixtures. These include solid supported lipid bilayers (SSBs) (Crane et al. 2005), free-standing planar bilayers (often called black lipid membranes or BLMs) (Collins and Keller 2008), inverted emulsion vesicles (IEVs) (Pautot et al. 2003), and large unilamellar vesicles subjected to cyclodextrin-mediated lipid exchange (LUVs) (Cheng and London 2011). The latter often make use of a dense vesicle core (typically a 20% sucrose solution) to aid vesicle purification by ultracentrifugation, although recent advances have eliminated this requirement (Heberle et al. 2016). Each type of model system has associated advantages and disadvantages, and a particular choice is often based on requirements of the measurement technique. For example, SSBs allow for facile measurement with surface sensitive techniques including AFM and sum-frequency generation (SFG) vibrational spectroscopy, while vesicles are particularly useful for ensemble-averaged spectroscopic and small-angle scattering measurements. It is also important to consider potential artifacts associated with the various model systems. For example, SSBs have an inherent propensity for asymmetric leaflet properties due to the close proximity of the support, which may alter lipid diffusion in the proximal leaflet (Scomparin et al. 2009; Sterling et al. 2015) and change the bilayer’s phase behavior (Goksu and Longo 2010; Seeger et al. 2010). Furthermore, the rapid equilibration of the two leaflets seen in some fluid phase SSBs may also preclude any meaningful study of asymmetry (Crane et al. 2005; Liu and Conboy 2005). BLMs and IEVs require the presence of long chain hydrocarbon solvents that can partition into the bilayer, and are known to influence bilayer thickness and thermotropic behavior (McIntosh et al. 1980). Our own modifications of the cyclodextrin-mediated exchange methodology, described below, were driven by a desire to eliminate sucrose from the LUV core, which we found could in some cases thin the bilayer due to osmotic stress (Heberle et al. 2016).

### Engineering asymmetric vesicles

London and co-workers pioneered the use of cyclodextrin for preparing freely floating asymmetric vesicles (Cheng et al. 2009; Cheng and London 2011). Cyclodextrins (CDs) are ring-shaped oligosaccharides—their outer surface is hydrophilic which renders the molecule water soluble, but the inner cavity is hydrophobic. CDs possessing six or more subunits have a cavity that is large enough to extract a hydrocarbon chain from the bilayer and partially shield it from water, making CDs effective catalysts for intervesicular lipid exchange through an aqueous solvent (Leventis and Silvius 2001). Asymmetric unilamellar vesicles (aLUVs) can thus be generated when two pools of vesicles having different composition are mixed in the presence of CD: sonicated or extruded *acceptor* vesicles provide lipids for the inner leaflet, while *donor* vesicles (typically MLVs) provide different lipids for the outer leaflet. To achieve a high degree of outer leaflet replacement, an excess of donor lipid (typically 2- to 3-fold over acceptor) is used. Exchange requires co-incubation and eventual separation of donor and acceptor vesicles, which is accomplished by density or size differences between the two vesicle pools. For example, by trapping a 25% (w/v) sucrose solution in the acceptor LUV core, aLUVs can be purified from large, low-density donor MLVs and CD by ultracentrifugation (190,000*g*) through a sucrose solution of intermediate density (Cheng and London 2011). Alternatively, sucrose-free aLUVs can be prepared by instead trapping sugar between the lamellae of the donor MLVs (Heberle et al. 2016), thereby enhancing the difference in sedimentation velocities of donor and acceptor vesicles in water, due to both the larger size and density of donor MLVs. In this case, low-speed centrifugation (20,000*g*) is sufficient to purify the aLUVs. Subsequent cycles of dilution and concentration using centrifugal ultrafiltration devices can then be used to efficiently remove residual sucrose and CD, and exchange H_2_O with D_2_O for SANS and ^1^H NMR measurements if desired.

Following aLUV purification, the individual leaflet compositions can be determined with high precision. First, the overall aLUV composition is measured with gas chromatography (GC) coupled to mass spectrometry (MS), following acid methanolysis to convert the chains to fatty acid methyl esters (FAMEs). This analysis works whenever there is a difference in the chain structure (number of carbons, number or type of double bonds) or isotopic content (deuterated vs. protiated chains) of the donor and acceptor lipids. Alternatively, ultra-performance liquid chromatography (UPLC)/MS has been applied to determine lipid exchange by mass (Eicher et al. 2017). Second, ^1^H NMR can be used to determine the transbilayer distribution of a headgroup-protiated lipid in a headgroup-deuterated lipid background after external addition of the paramagnetic shift reagent Pr^3+^, which selectively shifts resonances of outer leaflet lipids (Heberle et al. 2016; Marquardt et al. 2017). This assay is specific to lipids with a protiated choline headgroup (i.e., it works for any PC or SM lipid). The information from the GC/UPLC and NMR assays can then be used to calculate the inner and outer leaflet compositions (Heberle et al. 2016).

### Structure of asymmetric vesicles

A major challenge in membrane biophysics is to determine how the properties of the two leaflets of an asymmetric bilayer are coupled. Scattering techniques are important in this regard, as they in principle allow a comparison of leaflet structural properties (e.g., area per lipid and thickness) in an asymmetric bilayer, compared to the same composition in a symmetric bilayer. An advantage of scattering techniques (and especially SANS) is the ability to generate contrast between the two leaflets, and moreover to generate differently ‘visible’ aLUVs that nevertheless have identical structure, thereby enabling a joint analysis and improving the probability of a unique structural analysis of each leaflet. This is especially important for asymmetric bilayers, where the number of model parameters necessary to describe the transverse structure is effectively doubled relative to a symmetric bilayer. Equally important is the ability to constrain some structural parameters with independent measurements, for example, lipid molecular volumes from densitometry experiments as described in an earlier section. In the case of asymmetric bilayers composed of two lipids, the compositions of each leaflet can be independently determined provided one can measure both the overall composition of the vesicle, and the transbilayer distribution of one of the lipids, for example, using chromatography and ^1^H NMR as described in the previous section.

**Fig. 8:**
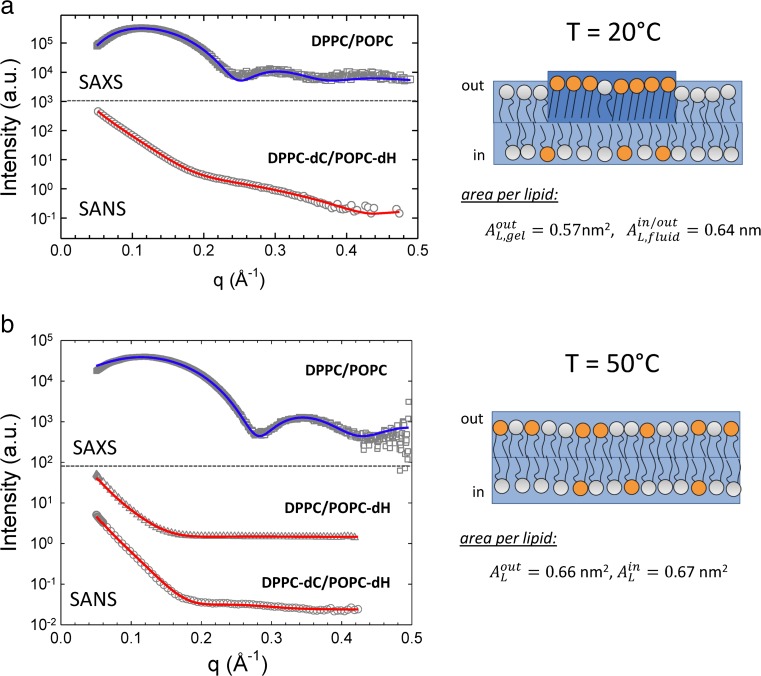
Structure of DPPC/POPC aLUVs above and below the melting temperature of DPPC via a joint analysis of SAXS and SANS data using headgroup (*dH*) and hydrocarbon (*dC*) deuterated lipid variants. At low temperatures (**a**) DPPC-rich gel-like domains coexist on the outer leaflet with a fluid POPC-rich phase. The area per lipid is significantly larger than in symmetric DPPC bilayers (0.47 nm^2^). The gel-like domains melt upon raising temperature to 50 °C (**b**). The larger content of POPC on the inner leaflet leads to a slightly larger average area per lipid as compared to the outer leaflet. Figure adapted from Heberle et al. (2016) and Eicher et al. (2017)

Heberle and coworkers first reported the use of SANS to examine the structure of aLUVs (Heberle et al. 2016). Control experiments in which aLUVs having different isotopic variants of POPC in the two leaflets were jointly analyzed using a 4-slab asymmetric model, demonstrated that the vesicle structure was not altered by the exchange process. The authors also examined a chemically asymmetric LUV, in which DPPC was exchanged into the outer leaflet of POPC vesicles and measured at 20 °C. The resulting aLUV had an outer leaflet containing 34 mol% DPPC, and an inner leaflet composed of 98% POPC. Successful modeling of *I*(*q*) required the use of two asymmetric form factors, implying the presence of coexisting outer leaflet environments—a relatively more ordered DPPC-rich phase (*A*_*L*_ = 0.57 nm^2^) and a disordered POPC-rich phase (*A*_*L*_ = 0.64 nm^2^) (Fig. 8). Interestingly, the DPPC-rich phase was more loosely packed than typical gel phases, implying partial fluidization due to interleaflet coupling. More recently, Eicher et al. proposed a higher resolution structural analysis based on the SDP models described in a previous section (Eicher et al. 2017). A structural analysis of DPPC/POPC aLUVs indicated that increasing the temperature above the melting transition of DPPC eliminated the interleaflet coupling.

### Lipid flip-flop

**Fig. 9:**
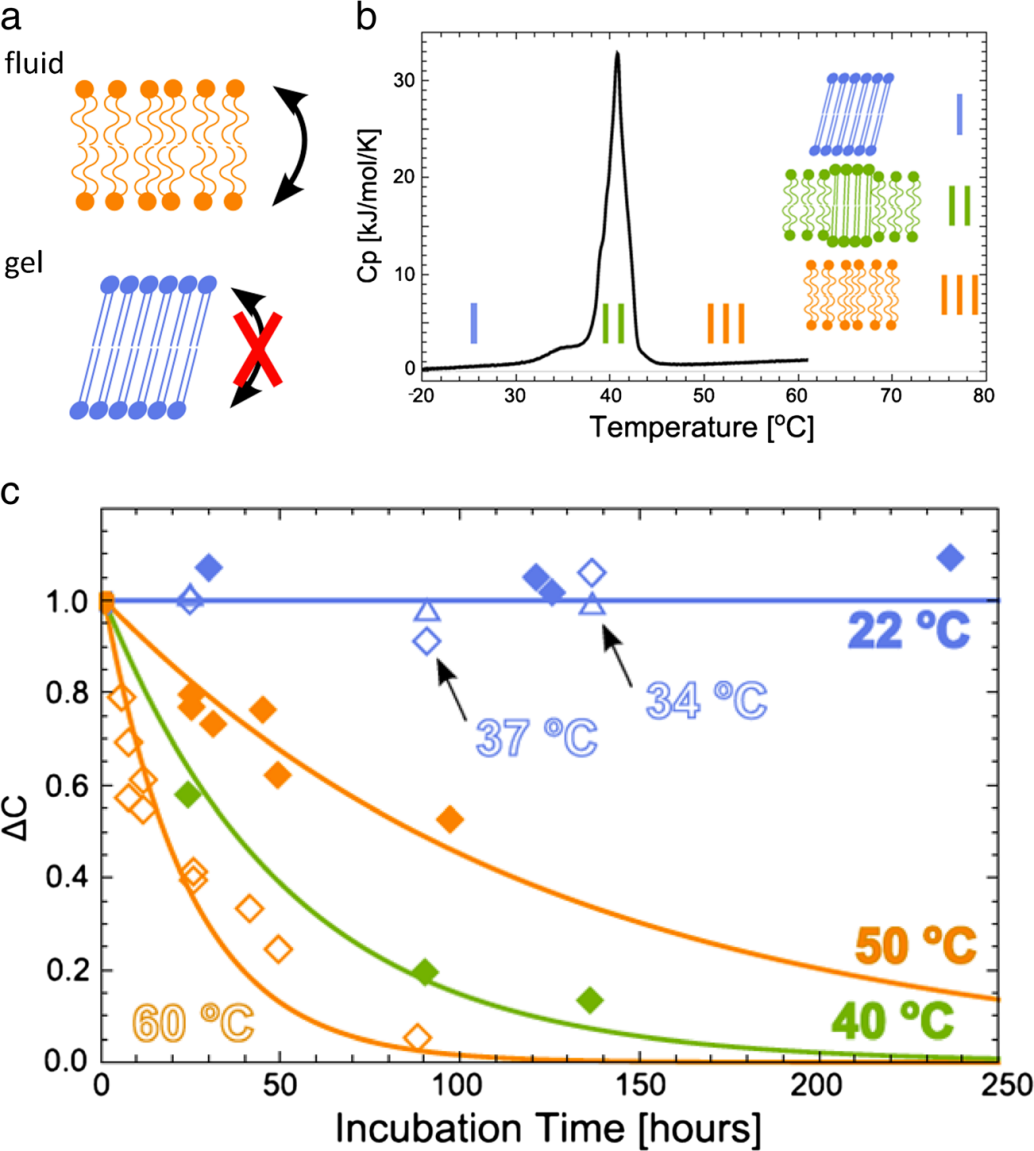
Passive lipid flip-flop as determined from solution NMR in isotopically asymmetric DPPC aLUVs. The schematic in (**a**) illustrates the main results: flip-flop half-times of days in the fluid phase and practically no flip-flop in the gel phase. **b **The specific heat capacity of the aLUVs as determined by calorimetry, indicating three main regimes for which flip-flop rates were determined (**c**). In regime I (gel), no flip-flop was detected within 250 h, increased flip-flop close to the phase transition (regime II) and a monotonous temperature dependent increase of flip-flop in fluid phase (regime III). Figure adapted from Marquardt et al. (2017)

As mentioned in the previous section, lipid asymmetry in vesicles can be monitored with ^1^H NMR, which allows a characterization of flip-flop kinetics without the need for extrinsic probes. Because flip-flop has historically been challenging to measure, there is little systematic information for how lipid structure (e.g., headgroup polarity and charge, chain length) affects translocation rates in vesicles. As summarized in Fig. 9, we recently measured flip-flop rates as a function of temperature for DPPC vesicles, finding flip-flop half-times are on the order of days in the fluid phase (*T* ≥ 50 °C), and were too slow to accurately measure in the gel phase (*T* ≤ 37 °C) (Marquardt et al. 2017). Moreover, flip-flop was accelerated for a sample incubated within the main phase transition at 40 °C. Interestingly, these findings were in contrast to SFG studies of solid-supported bilayers, where fast flip-flop half-times (minutes to hours) were reported for gel phase DPPC, and flip-flop was too fast to accurately measure for fluid phase DPPC (Liu and Conboy 2005; Anglin et al. 2010). The discrepancies between flip-flop in vesicles compared to SSBs may be related to a greater propensity for defect formation in some supported bilayers (Wu et al. 2016) suggested from MC simulations (Marquardt et al. 2017).

For future work, it will be critical to determine what factors influence the stability of asymmetric model membranes. Son and London found that shorter chain and polyunsaturated lipids present in the inner leaflet undergo relatively fast flip-flop in asymmetric vesicles having an ordered SM outer leaflet (Son and London 2013); for example, aLUVs prepared from DMPC acceptors were only weakly asymmetric, suggesting rapid flip-flop of this short (14 carbon chains) lipid when opposite the highly ordered SM lipid. Fast DMPC flip-flop was also observed in single-component DMPC vesicles using SANS (Nakano et al. 2009). However, a recent report of enhanced bending rigidity in DMPC/DOPC inverted emulsion GUVs implies a significant degree of asymmetry (and, hence, slow DMPC flip-flop) when DMPC is opposite a leaflet composed primarily of disordered DOPC (Lu et al. 2016a). Together, these results suggest that flip-flop rate is not an intrinsic property of a lipid but rather is context-dependent, changing with the chemical composition of the vesicle in which it resides (including differences in the compositions of the two leaflets).

## Coupling through the aqueous phase

As mentioned in the “Introduction”, the intracellular space is tightly packed with proteins, nucleic acids, carbohydrates, and membranes. Thus, heavy confinement is rather the rule than the exception on macromolecular length scales. This implies that generic intermolecular forces, in addition to specific (e.g., recognition) interactions, play a significant role for molecular assemblies. Moreover, these intermolecular potentials are modulated by the properties of the aqueous phase, a well-known example being simple Coulomb screening of charged surfaces by ions. However, thermally-induced bending fluctuations of soft membranes rapidly render what may appear a simple problem quite complex, as illustrated in our second example. First, we review domain interactions.

### Forces between like domains

One of the intriguing findings in MLVs displaying coexisting Lo + Ld domains is the long-range alignment of like-domains of micrometer sizes (Tayebi et al. 2012). This fact allowed us to disentangle transbilayer structural details of both domains using only SAXS (see above). The more fundamental question of why such alignment occurs may be relevant to domain–domain recognition processes between communicating cells, for example, during the T-cell-mediated immunological response (Monks et al. 1998; Grakoui et al. 1999).

**Fig. 10:**
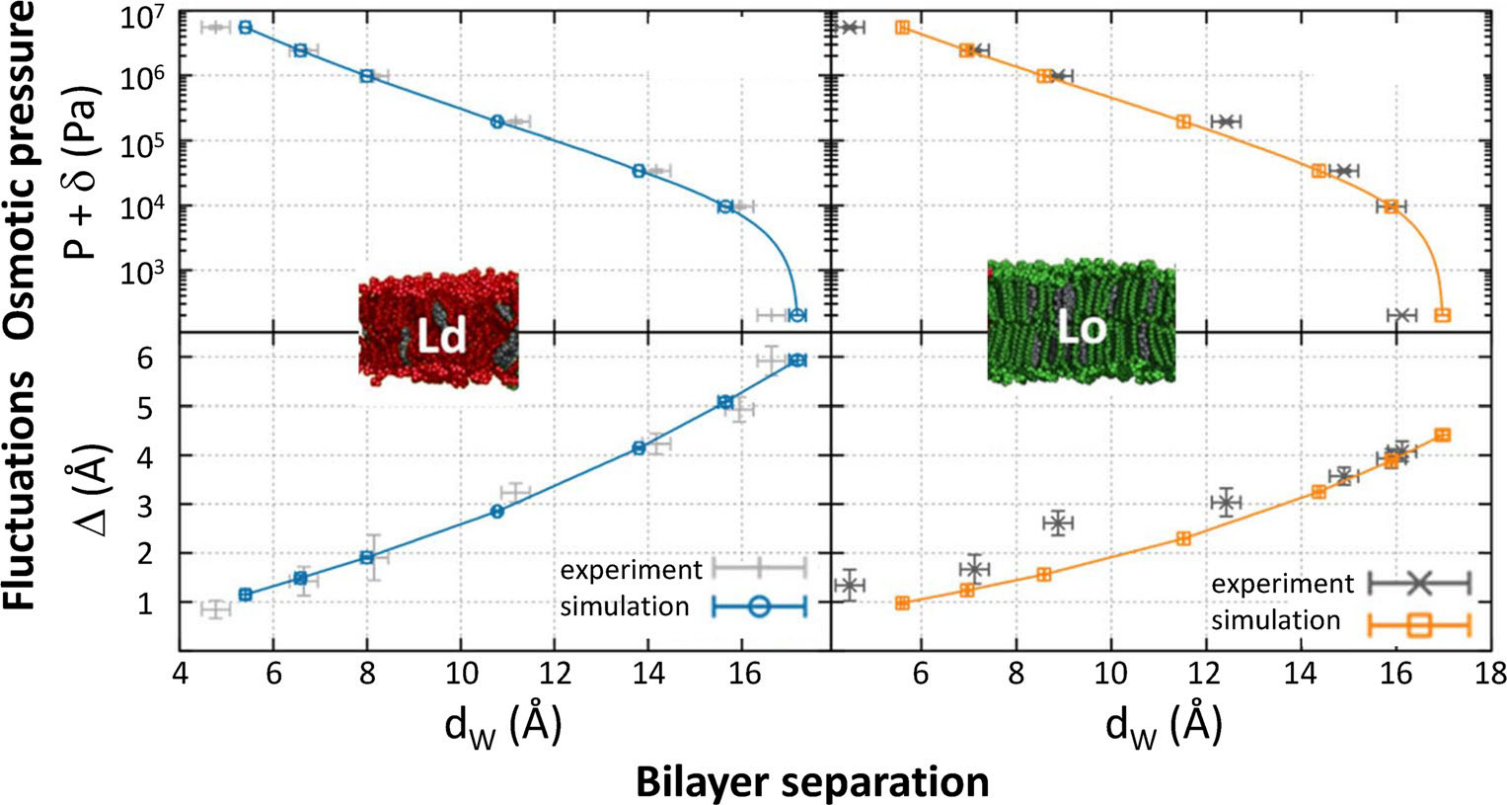
Analysis of osmotic stress data from MLVs displaying macroscopic Lo + Ld domains. Like-domains exhibit long-range positional alignment and behave independently when subjected to osmotic stress. The osmotic pressure over bilayer separation data was analyzed jointly with average membrane fluctuation amplitudes. Figure adapted from Kollmitzer et al. (2015)

In order to address domain alignment in multibilayers, we performed OS experiments on phase-separated MLVs (Kollmitzer et al. 2015). For each domain, we determined the osmotic pressure-dependent bilayer separation and bending fluctuations (Fig. 10). Due to charge neutrality, we could restrict our considered interactions to van der Waals attraction and hydration repulsion, both of which were coupled to MC simulations of an undulating stack of membranes. In this way, we could simultaneously fit bilayer separation and fluctuation data determined from SLDs and Bragg peak line-shape analysis. The result showed insignificant differences in van der Waals attraction between Lo–Lo and Ld–Ld domains. However, hydration forces decayed less rapidly with distance from the membrane surface for Ld domains, which were also about three times softer than Lo domains. Thus, a delicate balance of the latter two interactions appears to be the driving force for interdomain alignment. Support for this view has been given by a recent simulation study, which concluded that domain co-localization across the aqueous phase is facilitated by the differential undulations of Lo and Ld domains (Haataja 2017). The physiological role of such non-specific domain–domain interactions is currently unexplored, but may contribute to cell-to-cell communication and also to protein sorting and aggregation (see, e.g., also Monzel and Sengupta 2016).

### Effects of ions

Small ions (predominantly Na^+^, K^+^, Ca^2+^, and ATP) and macroions (e.g., proteins and DNA), which are abundant in the intracellular space, interact specifically with polar groups and modulate both short- and long-range intermolecular (intermembrane) interactions. These effects depend on ion charge, size and polarizability, which are commonly summarized as Hofmeister or ion-specific effects, respectively, and are present in any electrolyte solution (Kunz et al. 2004). However, it turns out that lipid bilayer properties in even simple electrolytes exhibit complex behavior, due to a coupling of all bare interactions (van der Waals, hydration and screened electrostatics) to entropically-driven membrane bending fluctuations.

**Fig. 11:**
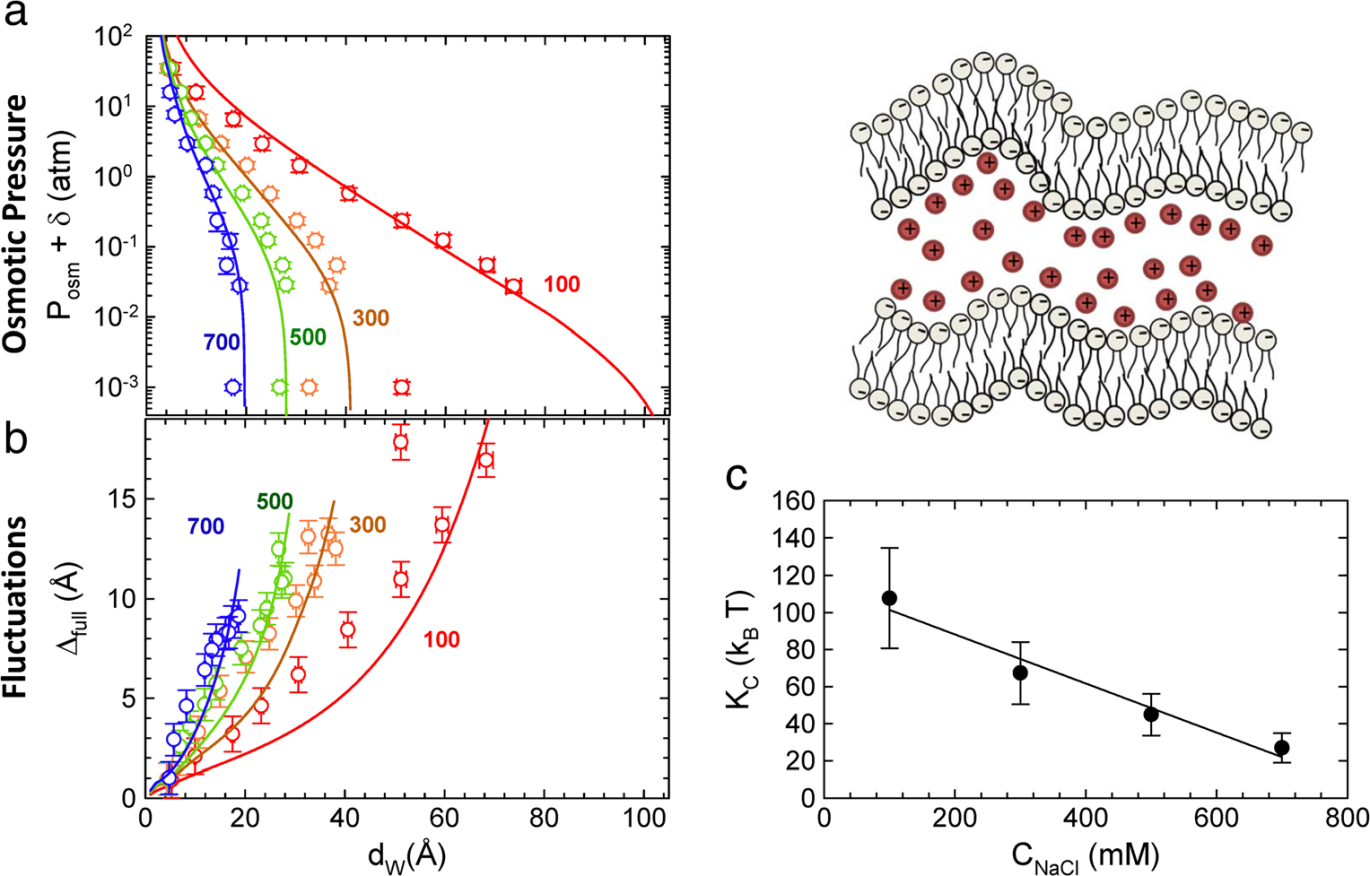
Effect of monovalent salt on interactions between charged lipid membranes. **a**, **b** The simultaneous analysis for OS data obtained for bilayer separation and undulations as a function of salt concentration (numbers adjacent to data give ion concentration in mM). **c** The ion concentration-dependent change of membrane rigidity. Figure adapted from Lu et al. (2016b)

Recently, we reported on the effect of NaCl on anionic lipid bilayers composed of dipalmitoyl phosphatidylglycerol (Lu et al. 2016b). As expected, increasing salt concentration screens the electrostatic repulsion between the charged membranes, leading to an observed decrease of their mutual separations. Interestingly, however, this effect couples to elastic properties of the membranes. Applying a theoretical framework that analytically combined screened electrostatic interactions within the Poisson Boltzmann mean-field approximation and other bare potentials with thermal bending fluctuations, we could account for the main features of corresponding OS data (Fig. 11). Results from this detailed analysis showed that the most prominent effect of increasing ion concentration is an almost 6-fold decrease of bending rigidity, while membrane structure was unaffected. Large bending rigidities of fluid charged membranes have been expected on theoretical grounds (Andelman 2006) and can be qualitatively understood by an in-plane stabilization of the bilayer due to charge repulsion.

These results clearly demonstrate the significant role of salts in modulating interactions and their coupling to membrane properties. Salt-specific effects are expected to be even more prominent for polyvalent ions such as proteins and DNA, strongly encouraging further exploration of such issues.

## Membrane-mediated coupling to transmembrane proteins

Lipid-protein interaction is a central and highly active field of biophysical research. Several excellent reviews have discussed the different variants of such interactions that can occur, including specific binding sites or recognition pockets on transmembrane proteins, annular lipids or unspecific, collective interactions, mediated by membrane elastic properties (Killian 2003; Lee 2004; Marsh 2008b; Paila et al. 2009; Baumgart et al. 2011; Smith 2012). Here, we focus on the latter mechanism, and more specifically on the effects of lateral pressure on protein partitioning into Lo + Ld domains.

**Fig. 12:**
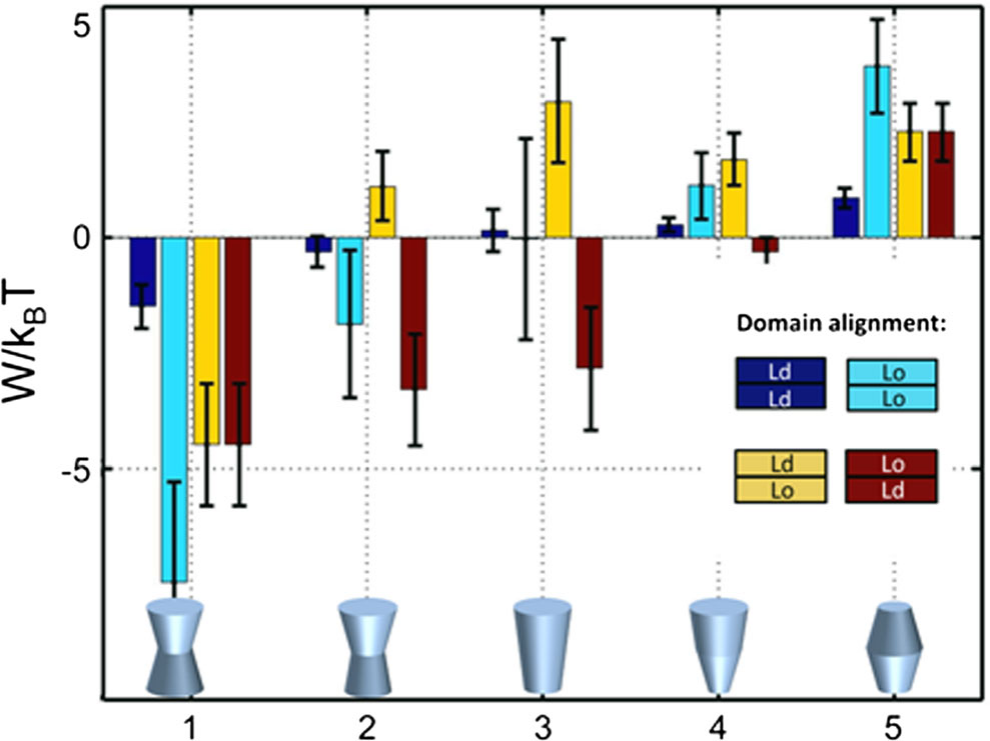
Lateral strain energy stored within either leaflet correlated or uncorrelated Lo/Ld domains for proteins of simple geometrical shape. For each protein shape, the domain configuration displaying the lowest energy is thermodynamically preferred. Figure taken from Frewein et al. (2016), with permission

Using bending rigidities, intrinsic curvatures and locations of the neutral planes determined for coexisting Lo + Ld domains in ternary mixtures of DSPC/DOPC/Chol (Kollmitzer et al. 2013, 2015; Heftberger et al. 2015), we were able to calculate the energetics and consequently the probabilities for protein partitioning in either domain (Frewein et al. 2016). In general, Lo domains are significantly more rigid and thicker than Ld domains, and also display larger intrinsic curvatures due to the higher amount of cholesterol (Kollmitzer et al. 2013). Here, we highlight our findings for proteins of either convex or concave shape, both of which were considered to be either symmetric or asymmetric with respect to the center of the bilayer (Fig. 12).

Our calculations showed that symmetric proteins of concave shape preferred a location in Lo domains due to tighter lipid packing, and do not prefer to form aggregates with other like-proteins. In contrast, symmetrically convex proteins preferentially partitioned into the more loosely packed Ld domains, and additionally favored the formation of protein clusters (Frewein et al. 2016). Proteins displaying asymmetry in shape with respect to the membrane center gave the lowest energies in leaflet anticorrelated domains. Shapes 2–4 shown in Fig. 12 preferred to be located in Lo^out^/Ld^in^ domains. Such findings correlated well with the experimentally observed partitioning behavior of the pentameric nicotinic acetylcholine receptor (nAChR), whose shape can be roughly described by a cone. For such shapes, our calculations showed similar energetic contributions in either leaflet correlated Lo or Ld domains (shape 3, in Fig. 12). This suggests no preference to be located in one of these domains, in excellent agreement with experiments (Bermúdez et al. 2010). Even more interestingly, a preferential location in Lo domains was observed in asymmetric vesicles enriched in sphingomyelin on the outer leaflet (Perillo et al. 2015). This agrees qualitatively with our calculations for leaflet-anticorrelated Lo/Ld domains (Fig. 12).

To summarize, these results show that there is an energetic contribution to protein orientation within the membrane that originates from transmembrane asymmetry. The commonly accepted positive-inside-rule (von Heijne, 2006), postulating a correlation between positively charged amino acid residues on cytosolic transmembrane protein domains and anionic inner leaflet lipids (PS, PI), certainly needs to be considered as well, for a more complete picture of protein topology. However, the other side of this coin is even more illuminating. Cells require transmembrane proteins with a fixed directionality, for example, pumping against solute concentration gradients. Thus, to have a properly working membrane protein machinery in cells, the orientation of the protein within the membrane needs to be fixed. In contrast, lipid translocation is energetically less costly, and may be facilitated at membrane defect sites as discussed above. Hence, depending on a protein’s shape, including grooves and polarity, it may ‘choose’ not only specific lipids in its immediate proximity (annular lipids) but also which lipids at some distance (‘bulk’) are preferred to yield the overall lowest free energy. It is easy to imagine how this could lead to membrane lipid asymmetry and the formation of lateral lipid gradients, i.e. rafts.

## Outlook: what next?

Research in membrane biophysics is as vibrant as it has ever been. With the rapid development of diverse experimental and theoretical tools, the field has recently gained insights from many different sides and views. SANS and SAXS can certainly claim an important role in this progress. Here, especially, advances in modeling scattering data, in combination with the creative use of contrast variation, have enabled the detailed analysis of complex biomimetic membranes exhibiting either domains of various size or an asymmetric distribution of membrane lipids. The latter development was tightly coupled to recent advances in engineering aLUVs as detailed above. While progress in understanding both symmetric raft-mimicking membranes and asymmetric membranes has been significant, it is also highly desirable that these two model systems are merged; the resulting hybrid model would represent a true first-order structural mimic of eukaryotic plasma membrane. Our report of asymmetric vesicles with domains in the outer leaflet (Heberle et al. 2016) is an important first step in this direction. Membrane asymmetry has also recently been shown to be of technological interest for diverse processes in drug delivery or biomedical imaging (Agudo-Canalejo and Lipowsky 2015).

While much work remains in lipid-only systems, there is also a need to increase the complexity of biomimetic membranes by including transmembrane proteins. Several groups have started exploring such systems by scattering techniques (see, e.g., Pan et al. 2009a; Skar-Gislinge et al. 2010; Denisov and Sligar 2016). At the same time, however, it is clear that increasing the level of complexity will lead to a decrease of details that can be modeled in SLDs. Otherwise, an inordinate number of parameters, even if extensive contrast variation is applied, may lead to results lacking physical meaning. However, such decisions need to be based on the given system of interest and can certainly not be generalized. A possibly tractable route is to couple scattering experiments on membranes to MD simulations, analogous to recent advances in protein solution scattering (Chen and Hub 2015). Much could also be learned by combination with neutron reflectivity studies of planar bilayers, which have recently evolved to being able to address issues related to protein complex formation at high structural resolution (for recent review, see Nanda et al. 2015).

Finally, in addition to the bottom–up approach discussed in this review, scattering experiments on live cells are increasingly being explored (see, e.g., Von Gundlach et al. 2016). While the accessible molecular detail is limited in these systems, such endeavors may be crucial in bridging research on well-defined model systems studied in equilibrium conditions to membrane structural roles in vivo.
